# Supplementary material for: Phenotypic Alteration of BMDM In Vitro Using Small Interfering RNA
Source: Cells. 2022 Aug 11;11(16):2498. doi: 10.3390/cells11162498 (PMC9406732; doi:10.3390/cells11162498)
Supplement: Supplementary file 1 [file cells-11-02498-s001.zip › cells-1841092-supplementary.pdf]

Supplementary Materials to Halimani et al, Phenotypic and functional alteration of BMDM in vitro using small interfering RNA

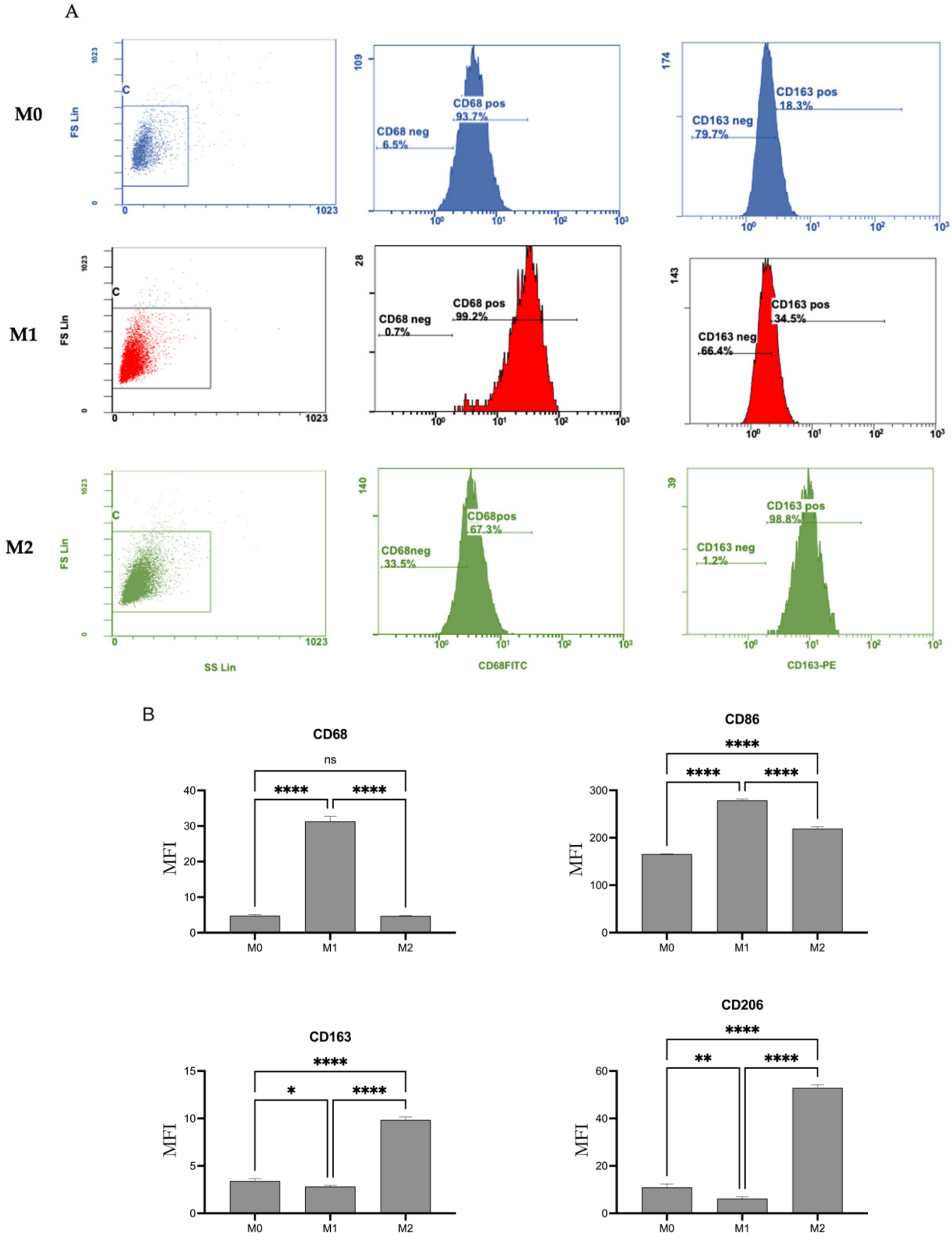

Figure S1. Polarization of BMDM. (**A**) Flow cytometric analysis showed high levels of CD163 in M2 macrophages (green panel) and high levels of CD168 in M1 macrophages red panel). M0 (blue panel) macrophages exhibited an intermediate phenotype. (**B**) The corresponding median fluorescence intensity (MFI) for cell surface antigens used to characterize polarization state. M1 macrophages exhibit high levels of CD68 and CD86 whereas M2 macrophages exhibit high levels of CD163 and CD206. Significance was determined by a one-way ANOVA at an overall p value of 0,05 followed by Turkey multiple comparison post hoc test: [$^*p < 0.05$; $^{**}p < 0.01$; $^{***}p < 0.001$; $^{****}p < 0.0001$]. Data is presented as mean±SD, n=3.

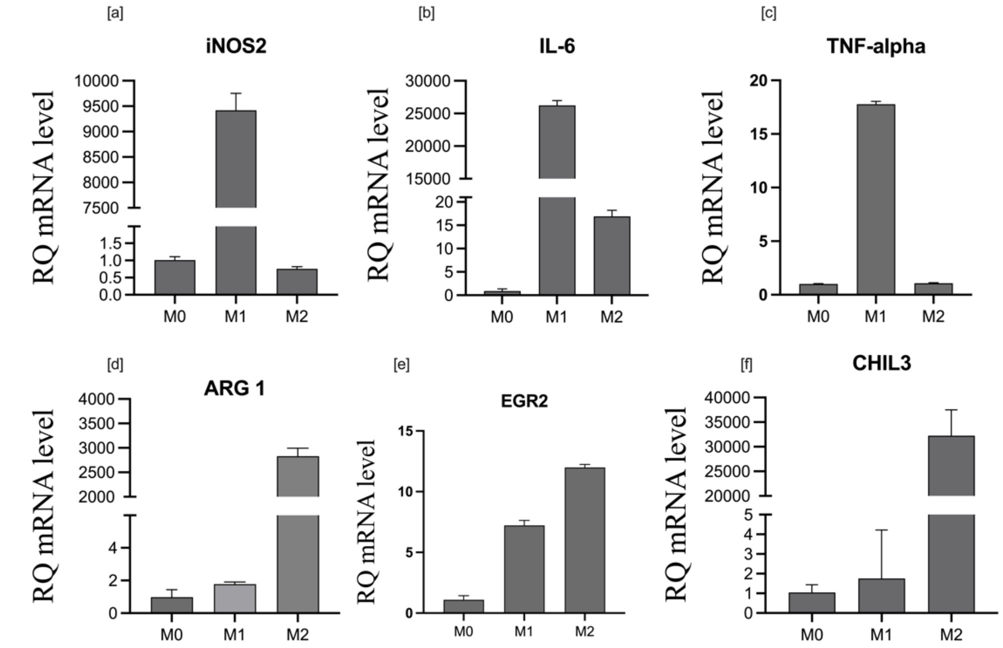

Figure S2. Polarization characterization in Raw 264.7 cells by qPCR. Relative mRNA quantity of NOS2[a], IL-6[b], TNF-α [c] arg1[d], EGR2[e] and CHI3L3 [f] normalized to GAPDH expression in cells activated for 24 hrs. with 100ng/ml LPS (M1) or 20ng/ml IL-4 plus 20ng/ml IL-10 (M2) Data presented as mean ± SD, n=3.

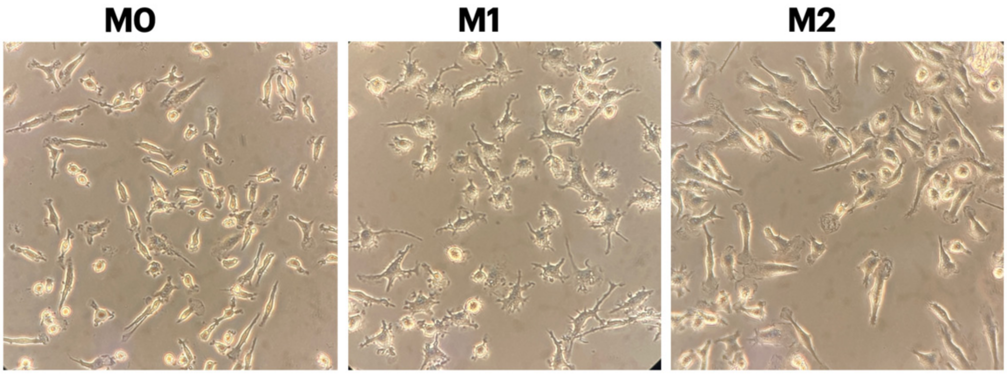

Figure S3. Polarisation-dependent morphological differences in murine M0, M1, and M2 macrophage cultures as visualised on a light microscope on 10x objective.

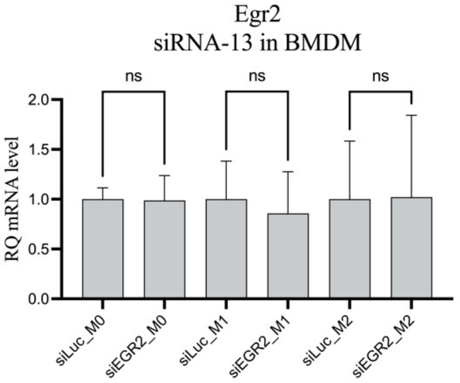

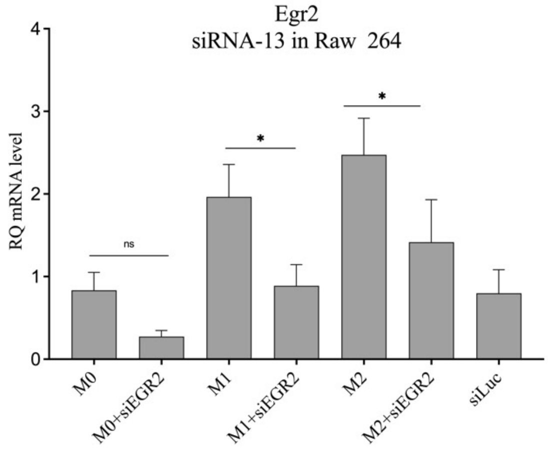

Figure S4. siRNA mediated knockdown of EGR2 in BMDM and Raw 264 cells by second siRNA sequence i.e., siRNA number 13, showing successful knockdown in polarised Raw 264 cells but no significant effect in polarised BMDM. Data presented as mean ± SD, n=3 [*p < 0.05; **p < 0.01; ***p < 0.001; ****p < 0.0001].

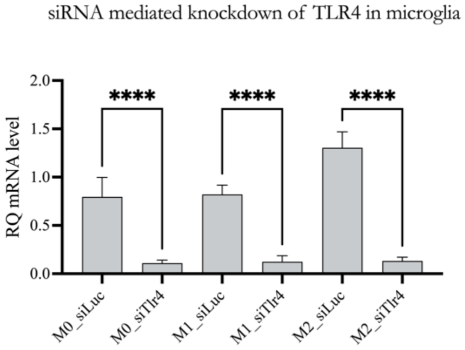

Figure S5. siRNA mediated knockdown of TLR4 in polarized microglia isolated from newborn pups. Data presented as mean ± SD, n=3 [*p < 0.05; **p < 0.01; ***p < 0.001; ****p < 0.0001].

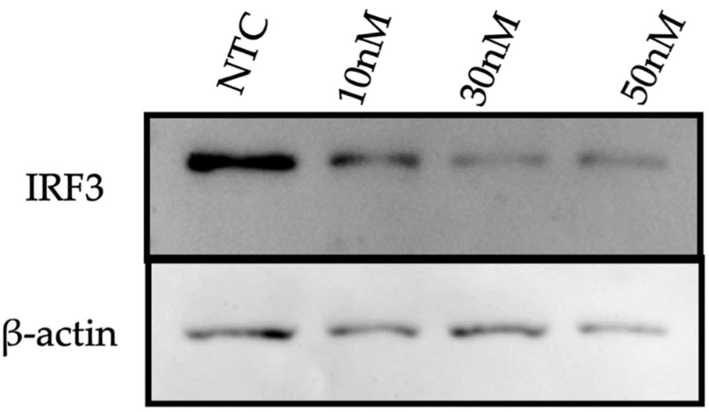

Figure S6. Western blot analysis of IRF3 in non-polarised BMDM after siRNA mediated knockdown of IRF3 for 7 days at varying 10nM, 30nM and 50nM siRNA concentration. [A]- IRF3 antibody [B]- Beta Actin antibody.

Table S1. Primer Sequence used for qPCR.

Gene Name	Sequence (F-Forward; R-Revers)
EGR2	F-GAGTGGCGGGAGATGGCA<br>R-AGGGTACTGTGGGTCAATGG
IRF5	F-TCCCAGACCCAAATCTCC<br>R-GTCCGTCAAAGGCAACAT
IRF3	F-ACCTACCGAAGTTATTTGATG<br>R-ACTCCCATTGTTCCTCAG
TLR4	F-TCCCTGATGACATTCCTTCT<br>R-GTTTCAATTTCACACCTGGAT AA
GAPDH	F-ACCTGCCAAGTATGATGA<br>R-GGAGTTGCTGTTGAAGTC
ARG1	F-AATTTACAAGACAGGGCTCC<br>R-GCATTCACAGTCACTTAGGT
IL-4	F-GCTAGTTGTCATCCTGCTCTTC<br>R-ATGGCGTCCCTTCTCCTG
NOS2	F-ACGTTTCTGGCTCTTGAG<br>R-CCTCTACATTTGCGGAGA T
IL-6	F-GACTGATGCTGGTGACAA<br>R-GCCATTGCACAACTCTTT
TNF	F-ACGCTCTTCTGTCTACTGAACTTC<br>R-GCTGCTCCTCCACTTGGT

Table S2. siRNA sequences for targets investigated.

EGR2s-6	AAUCuAAAUGGAuAuAcACTsT
EGR2as-6	GUGuAuAUCcAUUuAGAUUTsT
EGR2s-13	uAGGAcAuAUUGCUuAAGGTsT
EGR2as-13	CCUuAAGcAAuAUGUCCuATsT
IRF3s	AuGuGAAcAAcuuccuAAATsT
IRF3as	UUuAGGAAGUUGUUcAcAUTsT
IRF5s	AAGAAuGGccuGAuGucAATsT
IRF5as	UUGAcAUcAGGCcAUUCUUTsT
TLR4s	GcAuAGAGGuAGuuccuAATsT
TLR4as	UuAGGAACuACCUCuAUGCTsT
